# Identifying adult hypophosphatasia in the rheumatology unit

**DOI:** 10.1186/s13023-022-02572-7

**Published:** 2022-12-14

**Authors:** Julia Feurstein, Martina Behanova, Judith Haschka, Katharina Roetzer, Gökhan Uyanik, Benjamin Hadzimuratovic, Martina Witsch-Baumgartner, Georg Schett, Jochen Zwerina, Roland Kocijan

**Affiliations:** 1 Ludwig Boltzmann Institute of Osteology at Hanusch Hospital of OEGK and AUVA Trauma Centre Meidling, 1St Medical Department, Hanusch Hospital, Heinrich-Collin-Strasse 30, 1140 Vienna, Austria; 2 1St Medical Department, Hanusch Hospital Vienna, Vienna, Austria; 3 Vienna Bone and Growth Centre, Vienna, Austria; 4 Centre for Medical Genetics, Hanusch Hospital Vienna, Vienna, Austria; 5 Medical Faculty of Genetics, Sigmund Freud University, Vienna, Austria; 6 Division Human Genetics, Department of Medical Genetics, Molecular and Clinical Pharmacology, Medical University Innsbruck, Innsbruck, Austria; 7 Department of Internal Medicine 3, The University Clinic of Erlangen, Friedrich-Alexander-University Erlangen-Nuremberg, Erlangen, Germany; 8 Medical Faculty of Bone Diseases, Sigmund Freud University, Vienna, Austria

**Keywords:** Hypophosphatasia, Arthralgia, Alkaline phosphatase, ALPL gene, Musculoskeletal pain

## Abstract

**Background:**

The most frequent manifestation in adult hypophosphatasia (HPP) is musculoskeletal pain. The unspecific nature of its clinical presentation may prevent correct diagnosis. The aim of the study was to assess the prevalence of *ALPL* mutations in adult patients treated in rheumatological outpatient facilities with evident musculoskeletal symptoms typical for HPP.

**Methods:**

Over a period of 10 years 9,522 patients were screened in the rheumatology outpatient clinic of the Hanusch hospital Vienna. Serum ALP levels ≤ 40 U/L were found in 524 patients. After screening for secondary causes, 73 patients were invited for clinical evaluation. Genetic testing was performed in 23 patients with suspected HPP. Logistic regression models with Firth penalisation were used to estimate the unadjusted and BMI-adjusted association of each clinical factor with HPP.

**Results:**

Mutations in the *ALPL* gene were observed in 57% of genetically screened patients. Arthralgia, fractures, and pain were the leading symptoms in individuals with *ALPL* mutation. Chondrocalcinosis (OR 29.12; 95% CI 2.02–1593.52) and dental disease (OR 8.33; 95% CI 0.93–143.40) were associated with *ALPL* mutation, independent of BMI. Onset of symptoms in patients with *ALPL* mutation was at 35.1 (14.3) years, with a mean duration from symptoms to diagnosis of 14.4 (8.1) years. Bone mineral density (BMD) and trabecular bone score (TBS) as well as bone turnover markers were not indicative for HPP or *ALPL* mutation.

**Conclusion:**

HPP can mimic rheumatologic diseases. Thus, HPP should be considered as a possible diagnosis in adult patients presenting with musculoskeletal pain of unknown origin in rheumatology outpatient clinics. In patients with persistently low ALP serum levels and unclear musculoskeletal pain, HPP as the underlying cause has to be considered.

## Introduction

Since the introduction of the specific enzyme replacement therapy asfotase alfa, awareness for hypophosphatasia (HPP) has increased. HPP is a hereditary metabolic bone disease caused by a loss of function mutation in the *ALPL* gene encoding for the tissue non-specific alkaline phosphatase (TNSALP) [1]. Clinical manifestation of HPP is heterogeneous. Its primary hallmark is hypomineralisation, which can lead to bone deformities, fractures, delayed fracture healing as well as premature tooth loss, especially in children and adolescents. Extra-skeletal manifestations such as pulmonary abnormalities, cerebral seizures, impaired motor skills or nephrocalcinosis occasionally occur [2].

In adults, the leading clinical manifestation of HPP is musculoskeletal pain, often mimicking rheumatologic diseases [3–6]. In the study of Genest et al. concerning adults with paediatric onset HPP, all patients reported a history of muscular, dental, or skeletal complaints as well as pain at baseline. Rheumatic manifestations were stated by 21% of patients, while no detailed information was given [7]. Anecdotally, chondrocalcinosis, enthesitis and arthralgia/arthritis were described to occur in patients with HPP [8]. In a global registry, pain, orthopaedic procedures, and recurrent fractures were the most common manifestations in adult HPP patients [9]. Notably, most HPP patients suffer from more than one clinical HPP manifestation including dental disease, fractures or pain [10], [11].

Diagnosis of HPP can be challenging. Persistently low alkaline phosphatase (ALP) is the serological hallmark of HPP. However, low ALP is a common and often overlooked phenomenon in clinical routine and low ALP levels are not pathognomonic for HPP. Eating disorders, thyroid dysfunction, Milk-Alkali syndrome, multiple myeloma, Celiac Disease, zinc deficiency, surgery as well as anti-resorptive therapy can cause hypophosphatasemia [12, 13]. In a study by Schmidt et al., the prevalence of low ALP in adults was 8.46% for values < 30 U/L and more than 9% for ALP < 40 (but > 30 U/L) [13].

In the event of low ALP activity, substrates of ALP, such as pyridoxal 5’-phosphate (PLP) accumulate [12]. Elevated PLP levels are indicative of HPP and can be used to differentiate from secondary reasons for low ALP levels [14, 15]. Therefore concomitant PLP measurement in individuals with low ALP and clinical symptoms of HPP has been recommended [13]. Other established bone turnover markers such as osteocalcin, procollagen type 1 N-propeptide (P1NP), tartrate-resistant acid phosphatase 5b (TRAP5b), or N-terminal telopeptide of type 1 collagen (NTx) are usually in normal range and therefore not useful for the diagnosis of HPP [16]. Bone mineral density (BMD) by Dual Energy X-ray Absorptiometry (DXA), which is considered the gold standard for diagnosis and follow up of osteoporosis does not reflect metabolism or severity of the disease in patients with HPP [16].

Misdiagnosis is common and the diagnosis of HPP is often delayed due to the heterogeneity of the disease, unspecific symptoms as well as a lack of awareness for low ALP values [9, 17].

The aim of this study was to assess the prevalence of *ALPL* mutation in adult patients treated in the rheumatologic outpatient unit with evident musculoskeletal symptoms typical for HPP.

The primary objective was to identify *ALPL* mutations in patients presenting with musculoskeletal pain but an inconclusive diagnosis.

The secondary objective was to investigate the clinical differences between patients with *ALPL* gene mutation and those without mutation.

## Patients and methods

In this prospective single-centre study, all patients who presented at the rheumatology outpatient unit of the Hanusch Hospital Vienna, a specialized centre for rheumatology and rare bone diseases, between 2008 and 2018 were retrospectively screened for ALP serum levels ≤ 40 U/L.

Patients with at least one low ALP measurement were further evaluated using patient charts. Only adult patients (age ≥ 18 years) were included. Patients who presented with musculoskeletal symptoms (arthralgia, myalgia, arthritis, enthesitis) were included. All patients with secondary causes for low ALP levels (including eating disorders, thyroid dysfunction, surgery, sepsis) or, if available, consecutively normal ALP levels were excluded. Patients who fulfilled these inclusion criteria were invited for clinical evaluation. Clinical features and demographic data were documented with an HPP-specific questionnaire. To exclude secondary causes for low ALP, other metabolic bone diseases and assess HPP comorbidities, detailed laboratory examinations were performed in all participants. The following parameters in venous blood samples were evaluated: blood count, kidney and liver parameters, alkaline phosphatase, calcium, ionised calcium, phosphate, thyroid markers, 25(OH)Vitamin D, albumin, bone turnover markers such as osteocalcin (OC) and serum beta-crosslaps (CTX), as well as intact parathyroid hormone (PTH).

If laboratory results showed consistently low ALP ≤ 40 U/L and patients had clinical features of HPP but (i) no definitive rheumatologic diagnosis or (ii) an inconclusive rheumatologic diagnosis or (iii) were non-responders to their treatment, genetic testing was initiated.

Written informed consent was obtained from all individuals prior to any study related procedures. The study was approved by the Ethics Committee of the City of Vienna (EK 18-239-VK).

Genetic testing was performed on DNA extracted from peripheral blood samples by use of standard methods (QIASymphony, QIAGEN). Coding regions of *ALPL*-gene were enriched using the TruSight One Expanded panel and sequenced on a NextSeq device (both Illumina). Sequence data was aligned against the reference sequence ENST00000374840.8 and variants detected in coding and flanking intronic regions were classified following the ACMG guidelines [18]. Mutations were classified as Class 5 (pathogenic), Class 4 (likely pathogenic), Class 3 (VUS—variant of uncertain significance), Class 2 (likely benign) and Class 1 (benign).

After receiving genetic results, the study population was divided in two groups (HPP and non-HPP) to compare their clinical characteristics, biochemical profile, and demographic data. In addition, HPP patients underwent dual energy X-ray absorptiometry (DXA, GE Healthcare Lunar Prodigy (GE Healthcare, Madison, WI, USA)) to assess bone mineral density (BMD) and trabecular bone score (TBS). DXA analysis was conducted using the software Encore 16 and TBS was analysed using TBS iNsight 3 software. No information on DXA or TBS were available for the non-HPP group.

### Statistical methods

Characteristics of patients were described using frequencies and percentages for categorical variables and means and standard deviation (± SD) or medians and interquartile ranges (IQR) for continuous variables. Normality of parameters distribution in HPP and non-HPP was assessed by the Shapiro–Wilk test. Differences between the two groups were evaluated with T-test/Mann Whitney U test for continuous variables and Chi-squared/Fisher test for categorical variables, as appropriate.

The association between clinical parameters and HPP was assessed with logistic regression using the Firth method [19]. This method provides bias-reduction for small sample size and yields finite and consistent estimates even in case of separation. Crude model and model adjusted for body mass index (BMI) were fitted by penalised maximum likelihood.

All computations were performed in SPSS version 26 [20] and in R version 1.3, package “logistf”[21].

## Results

In the initial screening phase, 9,522 patients from the rheumatologic outpatient unit were screened for ALP levels. In total, 524 patients had at least one ALP measurement < 40 U/L. The remaining 8,998 patients presented with a serum ALP level > 40 U/L.

After screening the medical records, 426 patients were excluded due to secondary causes for ALP, transient low ALP, or an absence of typical musculoskeletal symptoms. Nineteen patients were excluded, as no medical records were available. Six patients had died.

The remaining 73 patients were invited to undergo clinical examination. Of these, 29 patients consented to clinical re-evaluation. Six more patients were excluded because serum ALP was > 50 U/L in subsequent blood analysis. In total, 23 patients showed repeatedly low ALP levels, typical musculoskeletal symptoms, no secondary causes, and underwent genetic testing.

**Fig. 1:**
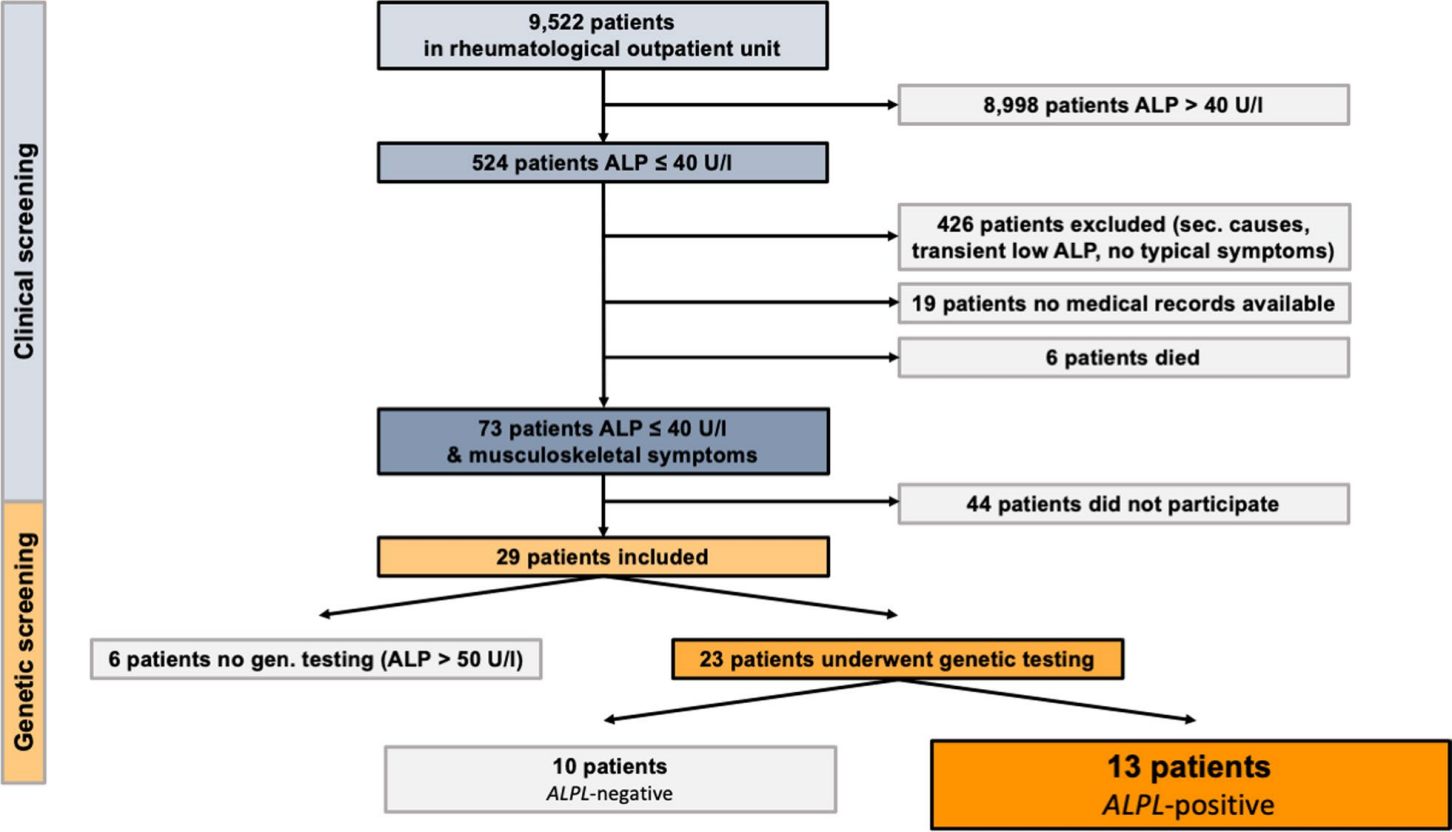
Study design. *ALP* alkaline phosphatase; *ALPL*-positive genetically confirmed mutation in the *ALPL*-gene; *ALPL*-negative negative genetic testing or ALP > 50 U/L

A mutation in the *ALPL* gene was observed in 13 patients (56.5%) (see Fig. 1). One patient was classified as Class 3 variant of uncertain significance but remained in the study due to typical clinical manifestations. Including only patients with Class 4 and 5 mutations, the prevalence of *ALPL* mutation was 52.2%.

Characteristics of patients with *ALPL* mutation and those without are presented in Table 1. Patients with *ALPL* mutation did not significantly differ in age, sex, or height from the control group without mutation. Mean age of heterozygous mutation positive individuals was 50.0 ± 14.2 (SD) with more women affected (61.5%). However, higher body weight (78.6 ± 19.9 kg vs. 62.0 ± 8.5 kg, *p* = 0.013) and BMI (27.5 ± 6.6 vs. 21.7 ± 2.1, *p* = 0.009) were found in *ALPL* mutation positive individuals. Patients with heterozygous mutation in the *ALPL* gene had significantly lower levels of ALP (27.7 ± 6.5 vs. 37.7 ± 7.2, *p* = 0.001) and higher vitamin D levels (89.0 ± 55.0 nmol/l vs. 78.0 ± 38.3 nmol/l, *p* = 0.045). No differences were found regarding calcium, phosphate, PTH, CTX or OC.

**Table 1:** Characteristics of patients with and without genetically confirmed HPP

Demographic data	*ALPL*-positive (n = 13) Mean (SD)	*ALPL*-negative (n = 16) Mean (SD)	*p*-value*
Age	50.0 (14.2)	48.7 (9.4)	0.767
Male (N. %)/Female (N. %)	5 (38.5)/8 (61.5)	2 (12.5)/14 (87.5)	0.104
Height (cm)	167.5 (10.8)	168.7 (10.0)	0.749
Weight (kg)	78.6 (19.8)	62.0 (8.5)	**0.013**
BMI	27.5 (6.6)	21.7 (2.1)	**0.009**
Alkaline Phosphatase (U/L)**	27.68 (6.51)	37.65 (7.19)	**0.001**
Calcium (mmol/l)	2.47 (0.085)	2.45 (0.075)	0.363
Ion. Calcium (mmol/l)	1.29 (0.030)	1.30 (0.035)	0.413
Phosphate (mmol/l)	1.20 (0.186)	1.09 (0.173)	0.097
Parathormone (pg/ml)	35.00 (12.34)	36.63 (7.40)	0.681
Beta-Crosslaps (ng/ml)	0.31 (0.15)	0.33 (0.13)	0.753
Osteocalcin (ng/ml)	17.47 (3.40)	18.76 (3.19)	0.303
25(OH) Vitamin D (nmol/l)	89.00 [55.00]	78.00 [38.30]	**0.045**
DXA L1–L4 (T-score)	0.063 (1.188)	NA	NA
DXA Femoral Neck (T-score)	− 0.700 (0.944)	NA	NA
DXA Total Hip (T-score)	− 0.675 (0.823)	NA	NA
TBS (L1–L4)	1.35 (0.099)	NA	NA

^*^Data expressed as mean ± SD or median [interquartile range]. *p*-values obtained by *t* test or Mann–Whitney U test, or Chi-squared test, as appropriate. *ALPL*-positive, genetically confirmed mutation in the *ALPL*-gene; *ALPL*-negative, negative genetic testing or ALP > 50 U/L; BMI, body mass index; DXA, dual energy x-ray absorptiometry; TBS, trabecular bone score; NA, not applicable

^**^Represents the mean value of multiple measurements

Bold indicates statistically significant findings

DXA measurements showed normal BMD values at the lumbar spine and hip as well as normal TBS values.

Logistic regression models with Firth penalisation adjusted for BMI showed that periarticular calcification was significantly associated with *ALPL* mutation (OR 29.12; 95% CI 2.02–1593.52). Similarly, patients with dental disease had a higher likelihood of *ALPL* mutation, although with borderline statistical significance (OR 8.33; 95% CI 0.93–143.40). A history of fractures, pneumonia, and impaired physical function in childhood were also more likely to be present in patients with *ALPL* mutation, albeit without statistical significance (see Table 2).

**Table 2:** Associations of clinical symptoms in individuals with genetically confirmed mutation in the *ALPL*-gene (*ALPL*-positive) and those without mutation (*ALPL*-negative) assessed by logistic regression with Firth correction

			Crude model (reference = non-HPP)		MI-adjusted model (reference = non-HPP)	
Symptoms	*ALPL*-positive (n = 13) N (%)	*ALPL*-negative (n = 16) N (%)	*p*-value	OR (95% CI)	*p*-value	OR (95% CI)
Fracture	10 (76.92%)	7 (43.75%)	0.079	3.80 (0.86–19.90)	0.990	1.01 (0.14–6.80)
Delayed fracture healing	1 (11.11%)	0 (0.00%)	NA	NA	0.927	0.83 (0.01–163.64)
Nephrocalcinosis	0 (0.00%)	1 (6.25%)	0.577	0.41 (0.00–8.46)	0.888	1.29 (0.01–35.06)
Pneumonia	6 (50.50%)	3 (18.75%)	0.090	3.86 (0.81–21.25)	0.080	4.85 (0.83–34.82)
Periarticular calcification	5 (38.46%)	1 (6.25%)	**0.040**	6.69 (1.08–73.59)	**0.010**	29.12 (2.02–1593.52)
Arthralgia	12 (92.31%)	15 (93,75%)	0.860	0.81 (0.06–10.87)	0.835	1.33 (0.08–26.01)
Muscle cramps	6 (46.15%)	8 (50.00%)	0.843	0.87 (0.21–3.60)	0.968	1.04 (0.18–6.60)
Pain in general	9 (69.23%)	9 (60.00%)	0.630	1.44 (0.32–6.83)	0.988	0.99 (0.17–5.73)
Epileptic seizures	0 (0.00%)	1 (6.25%)	0.543	0.38 (0.00–7.81)	0.969	0.93 (0.01–21.87)
Dental problems	5 (38.46%)	1 (6.25%)	**0.040**	6.69 (1.08–73.59)	0.058	8.33 (0.93–143.40)
Impairment in childhood	3 (23.08%)	1 (6.25%)	0.221	3.44 (0.48–39.45)	0.085	6.71 (0.78–94.71)
Skoliosis	4 (30.77%)	2 (12.50%)	0.247	2.75 (0.50–18.56)	0.934	0.90 (0.06–9.31)
Muscle weakness	6 (46.15%)	8 (50.00%)	0.843	0.87 (0.21–3.60)	0.878	1.14 (0.20–6.91)
Prior orthopedic surgery	4 (30.77%)	6 (37.50%)	0.724	0.77 (0.16–3.37)	0.266	0.36 (0.05–2.14)
Family history for HPP	0 (0.00%)	0 (0.00%)	NA	NA	NA	NA
Ability to walk	13 (100%)	16 (100%)	NA	NA	NA	NA

*ALPL*-positive, genetically confirmed mutation in the *ALPL*-gene; *ALPL*-negative, negative genetic testing or ALP > 50 U/L; OR, Odds Ratio; 95% CI, Confidence Interval; BMI Body Mass Index; NA, not applicable

Bold indicates statistically significant findings

Onset of symptoms was at 35.1 (SD 14.3) years, with a duration from symptoms to diagnosis of 14.4 (SD 8.1) years in heterozygous mutation positive individuals.

In 13 patients, rare genetic variants were identified of which 12 were classified as likely pathogenic or pathogenic (Class 4 or 5), one was classified as variant of unknown significance (Class 3) according to the ACMG guidelines [22]. All variants were in heterozygous state, which is in concordance with the mild clinical presentation of the patients (see Table 3).

**Table 3:** Genetical findings, initial diagnosis and main symptoms in *ALPL*-positive individuals

HPP	Mutation	Genetic condition	Class	Initial diagnosis	Main symptom
1	c.187G > C	Heterozygous	Class 5	Fibromyalgia	Pain
2	c.88C > T	Heterozygous	Class 5	Chronic back pain	Back pain
3	c.297 + 5G > A	Heterozygous	Class 3#	Unclassified myopathy*	Myalgia, arthralgia
4	c.1310C > T	Heterozygous	Class 4	Osteoarthritis	Arthralgia
5	c.542C > T	Heterozygous	Class 4	Pseudogout	Arthralgia
6	c.984_986delCTT	Heterozygous	Class 5	Tendovaginitis	Arthralgia
7	c.1490G > A	Heterozygous	Class 4	Calcific tendinitis	Arthralgia
8	c.797_802del	Heterozygous	Class 5	Gonarthrosis	Arthralgia
9	c.997 + 2 T > A	Heterozygous	Class 4	Fibromyalgia	Pain
10	c.1483G > A	Heterozygous	Class 4	Sjogren’s syndrome*	Arthralgia
11	c.526G > A	Heterozygous	Class 4	Spondyloarthropathy, seroneg. Rheumatoid Arthritis*	Back pain, arthralgia
12	c.283G > A	Heterozygous	Class 4	Spondyloarthropathy, sec. Fibromyalgia*	Back pain, arthralgia
13	c.984_986delCTT	Heterozygous	Class 5	Undifferentiated Connective Tissue Disease*	Myalgia

*Non-responder to disease-modifying anti-rheumatic drugs. #One patient was classified as class 3 variant of uncertain significance but remained in the study due to typical clinical manifestations

## Discussion

In the present study we investigated the prevalence of *ALPL*-mutations in a cohort of adult patients with musculoskeletal symptoms, typical for both HPP and rheumatological diseases. Screened patients were treated in the rheumatologic outpatient unit but had inconclusive diagnoses such as fibromyalgia, chronic back pain, pseudogout, calcific tendinitis, osteoarthritis, spondylarthopathy, or undifferentiated connective tissue diseases. Main symptoms were pain in general, back pain, arthralgia, and myalgia. Notably, several patients were treated with disease-modifying anti-rheumatic drugs and did not show any response to therapy. Genetic screening of our patients revealed a high prevalence of heterozygous mutation in the *ALPL* gene in those with persistently low ALP levels. Every second genetically tested patient showed a mutation in the *ALPL* gene, indicating a hereditary origin of the musculoskeletal symptoms. Low ALP was detected in 5% of our patient cohort and 14% of these patients presented with musculoskeletal complaints and repetitive low ALP compatible with a HPP diagnosis. Although only mild symptoms were detected in unselected populations with low ALP levels [23, 24] musculoskeletal pain, dental disease, and fractures were associated to *ALPL* variations [25]. Since musculoskeletal pain was an inclusion criterion in the present study, no significant differences were found between patients with and without *ALPL* mutation. However, periarticular calcification discriminated both groups. Moreover, fractures, pneumonia, dental disease, and impaired function in childhood were more common in heterozygous mutation positive individuals than in patients with low ALP, but without mutation in the *ALPL* gene.

Although the absolute number of patients with mutation in the *ALPL* gene identified in the entire study population is relatively low, it should be noted that HPP is a rare genetic disease with a low prevalence of 1/6370 for mild HPP [26]. In literature the prevalence of *ALPL* mutations depends on the investigated population and the geographic background [26, 27]. In an unselected population of adults from Spain with unexplained low ALP, *ALPL* mutations were found in 50% of patients. Musculoskeletal pain was again the predominant symptom in all patients with no difference between patients with and without genetic mutation [23]. In another Spanish cohort of more than 78,000 subjects, persistently low ALP was found in 0.12% of subjects. Fifty-six patients with low ALP and no secondary causes were identified. A mutation in the *ALPL* gene was found in seven out of 16 tested individuals. Most of them had the diagnosis of osteoarthritis, ankylosing spondylitis, fibromyalgia, or osteoporosis, respectively [24].

A comparable high prevalence of *ALPL* mutations were also found by others in more specific cohorts. More than 26,000 adults with endocrinological diagnoses were screened for ALP-levels in Denmark. Fifty-one subjects with persistently low ALP levels were identified after exclusion of risk factors for low ALP. Of these, 24 were genetically tested and more than 50% showed a mutation in the *ALPL* gene and musculoskeletal pain as leading symptom. PLP levels were higher in HPP than non-HPP subjects [15].

To date, there is only one study reporting on ALP levels in rheumatologic patients. Retrospective screening of medical records including existing genetical testing for *ALPL* gene mutation in adult patients with rheumatological diseases and repeatedly low ALP levels was done by a study group from Germany [28]. Persistently low ALP levels were detected in 1.31%, which is higher than reported in non-rheumatologic patients by others [24]. In 13 out of 19 previously genetically tested individuals (68.4%) a mutation in *ALPL* gene was found. Due to the nature of rheumatologic diseases and HPP, all subjects suffered from musculoskeletal pain. The most common rheumatologic diagnoses in *ALPL*-positive patients were osteoarthritis, rheumatoid arthritis, spondyloarthritis, and collagenosis. Chondrocalcinosis was not linked to HPP in this study [28]. It remains unclear if the patients in this and our study were misdiagnosed or *ALPL* gene mutation was a concomitant finding.

In contrast to the above-mentioned studies, other authors found a much lower prevalence of low ALP and *ALPL* mutations, respectively. In a Brazilian cohort of almost 290,000 chemical tests, only 12 patients with repeatedly low ALP and no secondary reason were identified. None of them showed typical symptoms such as musculoskeletal pain, fragility fractures, or chondrocalcinosis. Genetic testing was not performed. The authors hypothesised a lower HPP prevalence in Brazil compared to other countries [29].

In a paediatric setting, 393 out of 6,731 screened children had low ALP. The authors state that most of them had a feasible explanation. From the remaining 30 children only 3 had low ALP in combination with elevated PLP and ultimately an *ALPL*-mutation. One child was diagnosed with HPP, 2 were considered mutation carriers [30].

HPP is often overlooked in adult patients with typical manifestations. In adulthood, diagnosis is usually made above the age of 40 years. Whereas respiratory and dental symptoms usually occur in childhood, onset of pain and fractures was reported at the median age between 33 and 44 years [11]. In patients with first onset of symptoms in adulthood, symptoms preceded diagnosis by ten years or more [9, 25]. This is in line with our findings. Duration of musculoskeletal symptoms to detection of *ALPL* gene mutation was more than 14 years in our study. However, 23% of patients had also reported functional impairment in childhood pointing to the fact that HPP can remain undiagnosed for decades.

Although most adults do not qualify for enzyme replacement therapy with asfotase alfa, diagnosis of HPP in adults is still important, because an overlap to osteoporosis may occur. In case of uncertain bone metabolism, anti-resorptive drugs should be avoided. Bisphosphonates and denosumab suppress bone turnover and TNSALP activity and might lead to atypical femoral fractures [31]. More recent data suggest a rather lower risk in HPP patients with heterozygous mutation and lack of histological signs of osteomalacia [32].

Low ALP is the hallmark of HPP. Nevertheless, reference values for ALP depend on age and sex [33] and the lower limit of normal is often not stated in lab reports. Moreover, as low ALP is a common finding, secondary reasons must be ruled out. The measurement of natural substrates such as PLP or PEA is an important diagnostic step in patients with persistently low ALP-levels and typical symptoms. In case of repeatedly low ALP levels in combination with increased values for PLP and typical clinical signs such as musculoskeletal pain, HPP must be considered [13]. Due to the lack of accessibility no PLP plasma concentrations were evaluated in our study. Considering that, predictive models and machine learning algorithms identified HPP patients more accurately based on levels of ALP and PLP than ALP alone [34]. Established bone turnover markers are not indicative for HPP [16] and BMD by means of DXA is not necessarily reduced in HPP, as was shown in our population, and supported by other studies [35]. While not mandatory, genetic testing confirms the diagnosis of HPP because Sanger sequencing and MLPA allow the detection of 95% of all ALPL mutations [15, 36].

It has to be considered, that a mutation in the *ALPL* gene does not necessarily confirms the diagnosis of HPP. The combination of low ALP, musculoskeletal pain and a mutation in the *ALPL* gene can be both, mild HPP or a heterozygous carrier state with unspecific symptoms. Moreover, chronic pain, which was used as inclusion criterion in the present study, is a common finding also in the general population. International studies have reported a prevalence of chronic pain between 11% and 51.3% in population-based studies [37–40]. Especially in the absence of other skeletal and extra-skeletal manifestations such as recurrent fractures, atypical femoral fractures, tooth loss, chondro- or nephro-calcinosis, patients are more likely heterozygous mutation positive individuals, rather than HPP patients. Thus, diagnosis of HPP should be made with caution, in order to not overdiagnoses HPP.

In conclusion, a mutation in the *ALPL* gene should be considered as a possible explanation in adult patients presenting with musculoskeletal pain of unknown origin in rheumatology outpatient clinics. In patients with persistently low ALP serum levels and unclear musculoskeletal pain, HPP as the underlying cause has to be considered.

## Data Availability

The datasets generated and analysed during the current study are not publicly available, but are available from the corresponding author on reasonable request.
